# A multifaceted approach to a successful and sustainable hand hygiene campaign in a large tertiary academic medical centre

**DOI:** 10.1186/1753-6561-5-S6-P255

**Published:** 2011-06-29

**Authors:** O Yow, S Callery, M Vearncombe

**Affiliations:** 1 Infection Prevention & Control, Sunnybrook Health Sciences Centre, Toronto, Canada

## Introduction / objectives

Hand hygiene (HH) is essential to patient safety and is the single most important method to prevent the spread of infections. However, HH compliance of health care workers (HCWs) has been consistently low. In 2007, we implemented a comprehensive HH campaign, adapting the materials from the *Just Clean Your Hands* campaign of the Ontario Ministry of Health and Long Term Care (MOHLTC). This study examines the components of this campaign, its impact and its sustainability in a large 1185-bed tertiary referral teaching hospital.

## Methods

The comprehensive program has 2 phases: setup and maintenance. The setup phase includes obtaining strong commitment from hospital leadership, setting up alcohol-based hand rub at point of care, providing intensive education sessions to all areas of the hospital, establishing an audit and feedback process, and creating a poster campaign. The maintenance phase includes ongoing audit and feedback, analysis of data to direct program improvement and refreshing of promotional efforts.

HH audits of HCW-patient interactions were performed by trained auditors in all inpatient and long term care areas and higher risk out-patient areas using a standardized, validated audit tool.

## Results

Before the campaign, the overall hospital average HH compliance was 43%. Immediately after the pilot setup phase, HH compliance rapidly and significantly improved to 62%. After the full setup phase had been implemented, the HH increased to 72%. In the maintenance phase, the HH compliance has continued to increase to 81% over the subsequent 2 years. This improvement is statistically significant.

## Conclusion

A multifaceted campaign is effective in rapidly improving and sustaining HH compliance at a large tertiary academic hospital.

## Disclosure of interest

None declared.

